# Inhibition of osteoclastogenesis by opsonized *Porphyromonas gingivalis*

**DOI:** 10.1096/fba.2018-00018

**Published:** 2019-02-05

**Authors:** Subramanya N. Pandruvada, Jeffrey L. Ebersole, Sarandeep S. Huja

**Affiliations:** ^1^ Division of Orthodontics, College of Dentistry University of Kentucky Lexington Kentucky; ^2^ Center for Oral Health Research, College of Dentistry University of Kentucky Lexington Kentucky; ^3^ Present address: College of Dental Medicine Medical University of South Carolina Charleston South Carolina; ^4^ Present address: School of Dental Medicine University of Nevada Las Vegas Las Vegas Nevada

**Keywords:** bone loss, immune response, macrophages, osteoclast, periodontitis

## Abstract

A crucial step in the pathogenesis of periodontal disease (PD) is activation of osteoclasts (OC) by numerous virulence factors produced by *Porphyromonas gingivalis* (*Pg*). To understand pathogenesis of PD and the role of specific adaptive immune responses, effects of antibodies on *Pg*‐induced OC differentiation and function were investigated. Human peripheral blood‐derived monocytes were differentiated to OC in the presence or absence of: (a) *Pg*; (b) antibodies to *Pg*; and (c) antibody‐opsonized *Pg*. Findings suggest significant induction of osteoclastogenesis by *Pg* when compared to control cultures, whereas opsonization decreased osteoclastogenesis by 45%. Immune receptor gene expression profile in the presence of opsonized *Pg* showed marked upregulation of TLR1 (three‐fold) and TLR2 (twofold) along with FcγRIIB (two‐fold) and FcγRIII receptors (five‐fold), but not TLR4 and FcRγ receptors. Interestingly, blocking FcγRIIB, but not FcγRIII receptor, reversed the inhibitory effects of opsonized *Pg* suggesting a critical role played by FcγRIIB in osteoclastogenesis. Furthermore, opsonized *Pg* transformed OC precursors to a “macrophage phenotype” suggesting a bone protective role of the immune complexes in modulating osteoclastogenesis, probably by competing as an agonist for pattern recognition receptors, and inducing selective activation of FcγRs with simultaneous suppression of FcRγ which regulates bone resorptive process. Further defining effective antibody isotypes, avidity, and antigenic specificity could improve targets for eliciting protective immunity.

**Abbreviations**
CFU: colony‐forming units
IC: immune complex
M‐CSF: macrophage colony stimulating factor
OC: osteoclast
PD: periodontal disease
Pg: Porphyromonas gingivalis
PRR: pattern recognition receptor
RANKL: receptor activator of nuclear Factor‐kappa B ligand
Sg: Streptococcus gordonii
TLR: Toll‐like receptor
TRAcP: Tartrate‐resistant acid phosphatase

## 1 INTRODUCTION

Periodontal disease (PD) is a polymicrobial biofilm‐induced inflammatory disease, responsible for the most frequent cause of tooth loss in adults.^1^ Periodontitis results in progressive destruction of the periodontal attachment apparatus and bone destruction due to host inflammatory responses.^2^, ^3^ *Porphyromonas gingivalis* appears to represent a chronic opportunistic infection with its pathogenic potential expressed in susceptible hosts. Numerous virulence factors derived from *Pg* are responsible for enhanced colonization, stimulation of destructive inflammatory responses, and suppression of host protective responses.^4^ Chronic infection and associated clinical features of PD have been shown to elicit local and systemic antibodies in humans that react with an array of antigenic components of *Pg*.^5^ Substantial evidence indicates that these antibodies provide protection to some extent in animal models and in humans, controlling the chronic infection.^6^, ^7^ The titer and antigenic specificity of these antibodies have been identified in numerous cross‐sectional and longitudinal investigations of human periodontitis.^8^ Translating the role of antibodies using murine, canine, and nonhuman primate models of disease showed a generally positive impact of active vaccination on disease outcomes initiated by *Pg *infection.^8^, ^9^ While active immunization with other oral bacteria appeared somewhat less effective in affording protection, these types of translational studies have not adequately addressed: (i) the antigen(s) that are specifically targeted by the effective antibodies, and (ii) the exact mode of action of the antibodies in providing the protection.

Fewer studies have indicated that sera from patients with periodontitis contain antibodies that promote opsonophagocytosis of *Pg* by neutrophils.^10^, ^11^ Opsonization or immune complex (IC) formation aid in recognition of pathogens by phagocytes for optimal host responses. These ICs regulate immunogenic responses via Fc receptors for IgG (Fc Rs) on hematopoietic cells.^12^, ^13^ Recent studies suggest that unopsonized *Pg *can be phagocytized by macrophages,^14^ and antibody‐Fc receptor (FcR) interactions are important for optimal phagocytosis and opsonization for the clearance of *Pg*, as it can evade host response by manipulating complement and toll‐like receptor (TLR) signaling to promote a nutritionally favorable inflammatory response.^15^ The subgingival environment support these nutritional requirements as inflammation serves the nutritional needs of asaccharolytic dysbiotic communities through the release of tissue breakdown products including peptides and heme‐containing compounds.^16^ Active immunization can ensure immune responses that can boost opsonization and effective clearance of pathogens. However, to even consider a next step regarding therapeutic vaccination against *Pg* for humans, we must better understand the underlying protective mechanisms of the antibody in altering the disease trajectory. Evidence exists describing that *Pg *and other oral pathogens can enhance osteoclast function.^17^ However, little is known regarding the activation/inhibition of OC by ICs that are generated in response to infection or immunization and the resulting effect on PD. Here, we address the capacity of ICs to modulate osteoclast differentiation and function by recapitulating adaptive immune responses in vitro that would impact alveolar bone homeostasis.

## 2 MATERIALS AND METHODS

### 2.1 Bacterial cultures

*Porphyromonas gingivalis* FDC381 and *Streptococcus gordonii* (*Sg*) ATCC 10558 were cultured in brain heart infusion (BD and Company, MD, USA) medium supplemented with 5 μg hemin/mL and 1 μg menadione/mL under anaerobic conditions (85% N_2_, 10% H_2_, 5% CO_2_) at 37°C.^18^ For formalin fixation, *Pg/Sg* were harvested by centrifugation (13,000 *xg*; 20 minutes) and washed in PBS‐EDTA (0.02 mol/L phosphate containing 1 mmol/L EDTA). Bacteria were fixed in 0.5% buffered formal saline by incubation at RT overnight.^19^ Once the cells have been fixed, they were washed extensively in PBS‐EDTA and stored at 4°C in a concentrated form. Heat‐killed *Pg* were prepared following incubation of cultures at 70°C in water bath for 1 hour, pelleted and stored in PBS at 4°C until usage.^20^ For live *Pg *treatments, the bacteria were harvested, centrifuged, and washed in PBS. The bacterial counts (colony‐forming units/mL; cfu) were determined by measuring the optical density (A_580_) and extrapolated using a standard curve.

### 2.2 Human sera and generation of immune complexes

Human sera derived from a biospecimen repository in the University of Kentucky Center for Oral Health Research,^21^ classified based on *Pg *IgG levels in sera as: Low (LO) = ~6.5 µg/mL and High (HI) = ~133 µg/mL corresponding approximately to those identified in periodontally healthy and severe periodontitis patients, respectively. To generate ICs, purified IgG to *Pg *from patient sera at 2 µg (LO; dilution from Low IgG pool) or 40 µg (HI; dilution from High IgG pool) are added to formalin fixed *Pg *(10^6 ^cfu) (antigen) and incubated for 1 hour at 37°C in vitro. These ICs or “opsonized *Pg*” were introduced to receptor activator of nuclear Factor‐kappa B ligand (RANKL)‐primed pre‐osteoclast cultures. Since the bacteria used in challenging OC were formalin‐fixed, no azide was used in the antibody preparation.

### 2.3 Osteoclast preparation and culture

Pre‐osteoclasts were prepared from human peripheral blood obtained from flushing leukoreduction filters obtained from the Kentucky Blood Center using established convention (Institutional Biosafety Committee at UK #B14‐2491‐M). Briefly, leukodepletion filters obtained from healthy donors were flushed to obtain trapped blood cells and purified on Ficoll followed by 50% Percoll gradients. Monocytes were purified from mononuclear cells (PBMC) by immunomagnetic depletion and employing a cocktail of monoclonal antibodies,^22^ cell purity was confirmed by assaying CD3, CD14, CD16, CD19 and CD56 signature by flow cytometry. Monocytes were plated in α‐MEM with 10% FBS, standard antibiotics, and key OC differentiation factors: macrophage colony stimulating factor (M‐CSF) (20 ng/mL) and RANKL (50 ng/mL) and then cultured until multinucleation (~2 weeks). The experimental design includes introduction of varying concentrations of *Pg *(10^4^, 10^6^, or 10^8^ cfu); antibodies to *Pg *~2 µg/mL (LO) or ~40 µg/mL (HI); and opsonized *Pg* (*Pg*LO/*Pg*HI) to define the antibody‐antigen relationship affecting osteoclast maturation and function. Endpoint analyses were done following formalin fixation of cultures and staining for tartrate‐resistant acid phosphatase (TRAcP) activity to score the effect of *Pg* on osteoclast differentiation.

For receptor neutralizing antibody experiments, monocytes derived from PBMC were cultured in the presence of M‐CSF and RANKL for 3 days (pre‐osteoclasts). Pre‐osteoclasts were incubated for 1 hour with 2 µg/mL neutralizing antibodies for FcγRII/FcγRIII/IgG isotype. Subsequently, *Pg *alone or opsonized (*Pg*LO/*Pg*HI) were introduced and cultured until multinucleation, fixed and stained for TRAcP, and quantified. Similarly, bone resorption assays were done by culturing OC for 2 weeks on bovine bone chips, in the presence of *Pg* or with opsonized complexes. At the end of the culture, cells were removed from bone surface by hypo‐osmotic lysis and mechanical brushing followed by Toluidine blue staining of the resorbed pits. Resorbed bone area was analyzed on acquired pit images using ImageJ. Normal human sera and purified human IgG antibody (Sigma‐Aldrich Cat# I4506, RRID:http://scicrunch.org/resolver/AB_1163606) alone served as controls. Data collection was blinded to the investigators to allow for an unbiased assessment of treatment effect.

### 2.4 Flow cytometry

Phenotyping of mononuclear cells was performed in 1% BSA and 3% human serum PBS according to standard methods using a panel of antibodies directed against monocytes, T‐ and B‐lymphocytes, NK cells, and erythrocytes. The following conjugated antibodies were used: anti‐CD19 (Beckman Coulter Cat# IM1284U, RRID:http://scicrunch.org/resolver/AB_131011), anti‐CD56 (Beckman Coulter Cat# IM2073U, RRID:http://scicrunch.org/resolver/AB_131195), anti‐CD3 (Beckman Coulter Cat# IM1282U, RRID:http://scicrunch.org/resolver/AB_10640418), anti‐CD14 (Beckman Coulter Cat# IM0645U, RRID:http://scicrunch.org/resolver/AB_130992), and anti‐CD16 (Beckman Coulter Cat# IM0814U, RRID:http://scicrunch.org/resolver/AB_10640417) were from Beckman Coulter (FL). FACS analysis was performed on a LSRII cytometer (BD Biosciences, CA). Mø phenotype was confirmed by flow cytometry targeting CD68 (R and D Systems Cat# IC20401P, RRID:http://scicrunch.org/resolver/AB_2074835) and CD80 (R and D Systems Cat# FAB140F, RRID:http://scicrunch.org/resolver/AB_357027), CD163 (R and D Systems Cat# FAB1607P, RRID:http://scicrunch.org/resolver/AB_2074536), and CD206 (R and D Systems Cat# FAB25342P, RRID:http://scicrunch.org/resolver/AB_10889015) antibodies all from R and D Systems (IN). Data were analyzed with Flowing Software (University of Turku, Finland) or FACSDiVa software (BD Biosciences) and represented, when required, with the logical display.

### 2.5 RT‐qPCR

PBMC‐derived monocytes were cultured in the presence of M‐CSF and RANKL for 7 days and treated either with *Pg* alone; antibodies to *Pg* (LO/HI); or with opsonized *Pg* (*Pg*LO/*Pg*HI) for 24 hours. Pre‐osteoclasts lysed in TRIzol (Invitrogen, CA) were processed for RNA and subsequent cDNA preparations using First Strand synthesis reagents (Roche). RT‐qPCR analyses were performed to assay immune receptor gene expression of TLR1, TLR2, TLR4, FcRγ, FcγRIIB, and FcγRIII genes (Table 1). In addition, typical OC marker genes (ACP5, RANK, NFAT, and CATK), macrophage markers (CXCL10, CXCL11, CCL17, CCL22, KLF4, and MRC1) were also assayed in the above treatment groups. Oligonucleotides were purchased from IDT‐DNA (IA). Concentration ratios of target genes were normalized to the S16 gene and gene expression levels were compared across the samples prepared from each of the treatment groups.

**Table 1:** Primer sequences

Gene	Primer sequence	
	Forward (5′‐3′)	Reverse (5′‐3′)
ACP5	AGG CTT TTC CTC CAA CCT GT	TTT CAC ATA CGT GGG CAT CT
CATK	CCG CAG TAA TGA CAC CCT TT	GGA ACC ACA CTG ACC CTG AT
CCL17	GGG TGT CTC CCT GAG CAGA	CAC ATT GGT CCC TCG AGC TG
CCL22	ATT ACG TCC GTT ACC GTC TG	TAG GCT CTT CAT TGG CTC AG
CXCL10	AGC AGA GGA ACC TCC AGT CT	ATG CAG GTA CAG CGT ACA GT
CXCL11	AGC AAG CAA GGC TTA TAA TCA AAA	TTG TTC TAG GTT TTT CAG ATG CCC T
FCGRIIB	TGAGTCCTGAAGCTCCCTGT	AGG TGC AGT CGG TTA TTT GG
FCGRIII	ACA GGT GCC AGA CAA ACC TC	TTC CAG CTG TGA CAC CTC AG
FCRG	TGA TTC CAG CAG TGG TCT TG	AGG AGG GTG AGG ACA ATT CC
KLF4	CCA TCT TTC TCC ACG TTC G	AGT CGC TTC ATG TGG GAG AG
MRC1	ACG GAC TGG GTT GCT ATC AC	TGA TCC CCA AAA GTG TGT CA
NFAT	TTT TCC TTG ATC CCT GTT GG	GCA GAA GAG CCA TGT TTT CC
RANK	GGC TTA CTA AAA CCG AGC TCA C	CAA ATG AAC GGT TGA CAC CA
S16	GTC ACG TGG CCC AGA TTT AT	TCT CCT TCT TGG AAG CCT CA
TLR1	GGG TCA GCT GGA CTT CAG AG	CGA ACA CAT CGC TGA CAA CT
TLR2	ATC CTC CAA TCA GGC TTC TCT	GGA CAG GTC AAG GCT TTT TAC A
TLR4	CCT CGG CGG CAA CTT CAT AA	AGA GCG GAT CTG GTT GTA CTG

### 2.6 Immunoblotting

Osteoclasts cultured for 10 days were treated with formalin‐fixed *Pg* or opsonized (*Pg*LO/*Pg*HI) for 48 hours. Whole cell lysates were prepared in RIPA buffer containing protease inhibitor cocktail following brief sonication (30 sec pulse, ×2). Equal protein concentrations were electrophoresed on 8%‐10% SDS‐polyacrylamide denaturing gels. After transfer to Immobilon‐P transfer membranes (Millipore, MA), blocked for nonspecific binding by incubating in 5% milk for 1 hour and with specific antibodies overnight at 4°C, over gentle agitation. Incubation in secondary antibodies was done for 1 hour in 5% milk before detection with an enhanced chemiluminescence procedure (Bio‐Rad, CA). Western blots were scanned and quantified by densitometry using ImageJ. The primary antibodies were used at: TLR2 (R and D Systems Cat# AF2616, RRID:http://scicrunch.org/resolver/AB_416645), TLR4 (R and D Systems Cat# AF1478, RRID:http://scicrunch.org/resolver/AB_354816) @ 1/2500 (R and D Systems, IN), FcγR IIB (R and D Systems Cat# AF1330, RRID:http://scicrunch.org/resolver/AB_354737), FcγR III (R and D Systems Cat# AF1597, RRID:http://scicrunch.org/resolver/AB_354882) @ 1/5000 and β‐Actin @ 1/10,000 (Santa Cruz Biotechnology Cat# sc‐47778 HRP, RRID:http://scicrunch.org/resolver/AB_2714189). The secondary antibodies (Santa Cruz, TX) were used @ 1/10 000 dilution.

### 2.7 Statistical analysis

Statistical evaluation of the data collected was applied to various phenotypic and functional markers for OC. The comparisons were derived from triplicate determinations of each cell preparation, prior to and after challenge with the *Pg *and antibody preparations. The data containing continuous variables, that is, the percentage of cells positive for TRAcP activity, pit area measurements, data obtained from mRNA expression levels, and protein expression from densitometric analysis of Western blots were compared among the conditions using ANOVA and the Tukey's post hoc multiple comparisons test (Graphpad software Inc, CA). An adjusted α level of 0.05 was accepted as significantly different.

## 3 RESULTS

### 3.1 *Porphyromonas gingivalis* stimulates osteoclastogenesis

**Figure 1:**
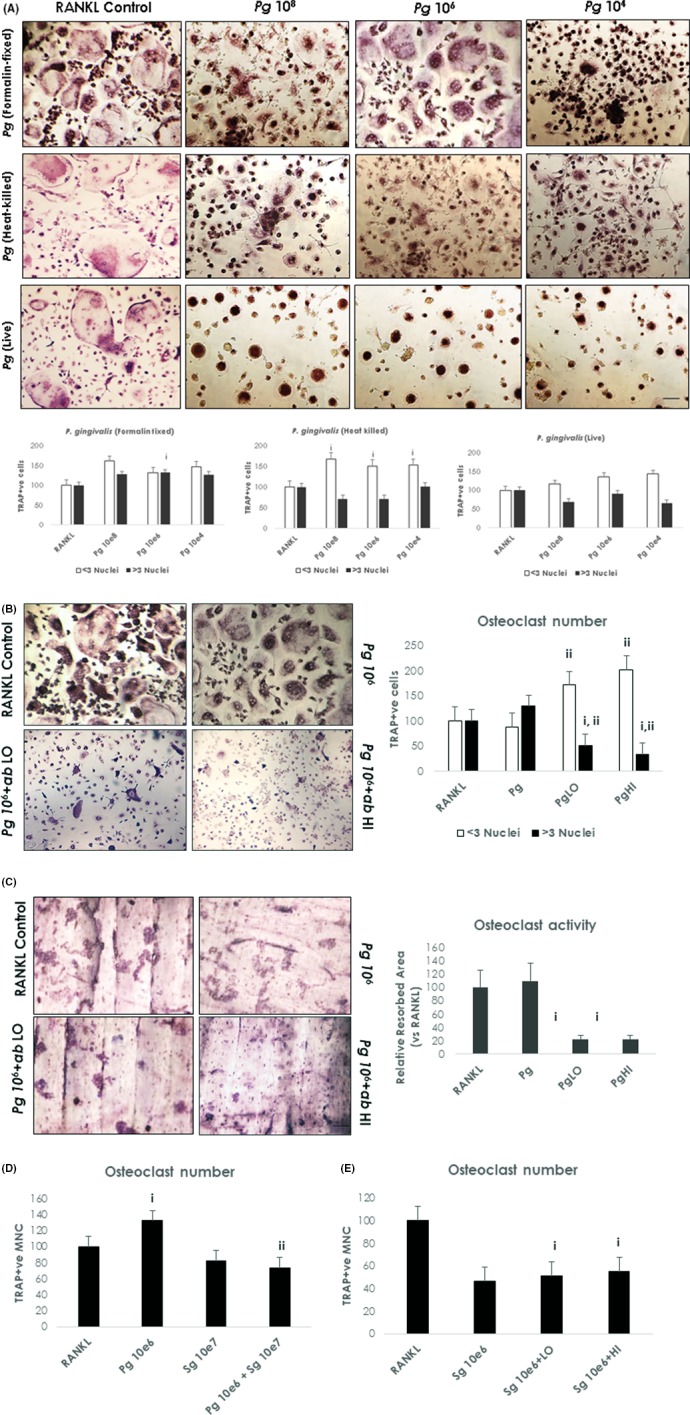
Opsonized *Porphyromonas gingivalis* inhibits osteoclast differentiation. A, Human peripheral blood‐derived monocytes from healthy donors were cultured in the presence of macrophage colony stimulating factor (M‐CSF) and receptor activator of nuclear Factor‐kappa B ligand (RANKL) or formalin fixed or heat‐killed or live *P gingivalis* at various cfu/mL for 2 weeks. Osteoclasts were stained for TRAcP activity, quantified, and grouped based on number of nuclei (three or more nuclei). Multinucleated cells with three or more nuclei are counted as osteoclasts and presented as a ratio over RANKL control. Data expressed as the mean ± SE of triplicate determinations from four independent donors. ^i^ *P* < 0.05 vs RANKL control. B, PBMC‐derived monocytes were cultured in the presence of M‐CSF and RANKL with or without opsonized bacteria (antibodies to *P gingivalis *complexed with formalin fixed*‐P gingivalis; Pg+abLO*/*Pg+abHI*) and *P gingivalis alone *(*Pg*) for 12 d. Osteoclast were fixed and stained for TRAcP activity and quantified. Note increased osteoclast number with *Pg* alone, whereas presence of antibodies to *Pg* abrogates this increase significantly. Data represent the mean ± SE from three independent cultures. ^i^ *P *< 0.05 vs RANKL control; ^ii^ *P *< 0.05 vs *Pg* alone. C, PBMC‐derived monocytes were cultured in the presence of M‐CSF and RANKL (RL CTRL) or *Pg* (10^6^) and/or with immune complexes (*Pg*+abLO/*Pg*+abHI) for 12 d. Osteoclast were removed by hypo‐osmotic lysis and pits were stained in Toluidine blue and were photographed. Pit area was measured using ImageJ. Histograms represent the percentage of resorbed area from three independent experiments. ^i^ *P *< 0.05 vs *Pg* alone. Scale bar, 200 µm. D, Human peripheral blood‐derived monocytes from healthy donors were cultured in the presence of M‐CSF and RANKL or formalin‐fixed *P gingivalis* (at 10^6^ cfu/mL); *Streptococcus gordonii* (10^10^ cfu/mL) or *Pg*+*Sg* combo for 2 weeks. Osteoclasts were stained for TRAcP activity and quantified. Histograms represent the percentage of MNC over RANKL group. Data expressed as the mean ± SE of triplicate determinations from three independent donors. ^i^ *P* < 0.05 vs RANKL control; ^ii^ *P* < 0.05 vs *Pg* alone. E, PBMC‐derived monocytes were cultured in the presence of M‐CSF and RANKL with or without opsonized bacteria (antibodies to *P gingivalis *complexed with formalin‐fixed *S gordonii; Sg+abLO*/*Sg+abHI*) and *S gordonii alone *(*Sg*) for 12 d. Osteoclast were fixed and stained for TRAcP activity and quantified. Note decreased osteoclast number with *Sg* treated cultures and no effect of opsonized *Sg* treatment. Histograms represent the percentage of MNC over RANKL group. Data expressed are the mean ± SE from three independent cultures. ^i^ *P* < 0.05 vs RANKL control

Human peripheral blood‐derived monocytes were primed with M‐CSF and RANKL for 3 days prior to the introduction of varying concentrations of *Pg *(either formalin‐fixed, heat killed or live form) and cytokines were renewed every third day until multinucleated OC appeared*.* Findings suggested a direct effect of *Pg *on OC with substantial enhancement of differentiation and numbers (Figure 1A). However, the presence of live *Pg *resulted in cell deterioration and death likely due to active bacterial secretion of deleterious factors, including LPS and gingipains.^23^, ^24^ As a starting point in the studies and given that formalin fixation would abrogate the impact of the proteases of *Pg*, while maintaining its general antigenic integrity based upon existing literature, formalin‐fixed bacteria were used in further experiments. As noted, this also enabled some standardization of challenge in treatment conditions. Follow‐up studies could use live bacteria, as well as strains lacking proteases, or accompanied by treatment with targeted protease inhibitors to explore their role in these critical processes. There is evidence to support that *Pg *modulates the commensal oral microbiota through host‐independent, direct effects in ways that are consistent with dysbiotic changes.^25^, ^26^ Thus, through both host modulation and direct effects on the microbiota, *Pg *may change its numbers and composition toward dysbiosis and accelerate bone destruction. Interestingly, the presence of an oral commensal bacteria, *Sg*, in cultures decreased *Pg‐*induced OC differentiation (Figure 1D).

### 3.2 Antibodies to *P gingivalis *alter osteoclast differentiation and function induced by *P gingivalis*

**Figure 2:**
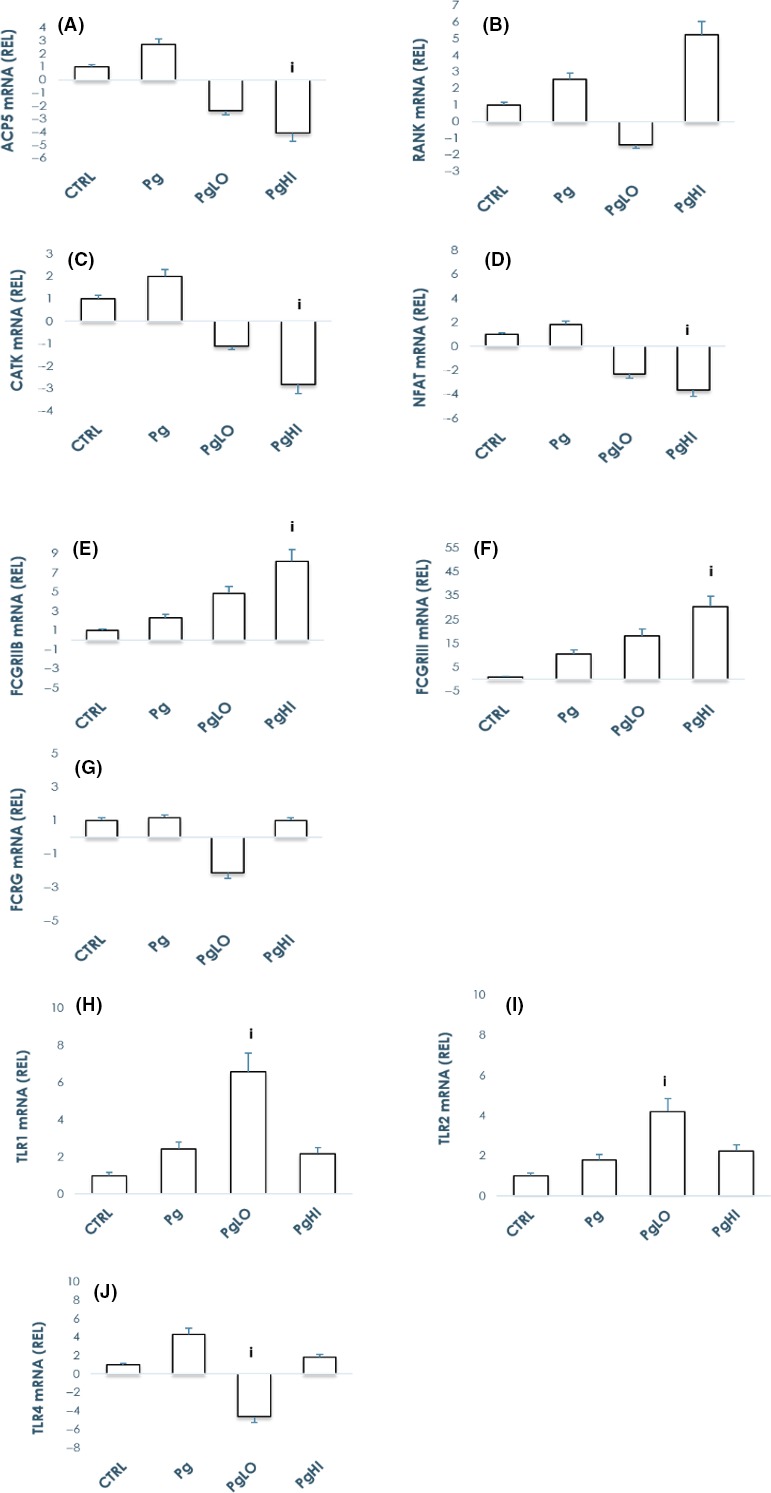
Opsonization of bacteria abrogates *Porphyromonas gingivalis*’ stimulatory effects by modulating immune receptors gene expression. PBMC‐derived monocytes were cultured in the presence of M‐CSF and RANKL for 7 d and treated with *Pg* or antibodies to *Pg* (LO/HI) alone or with opsonized bacteria (*Pg*LO/*Pg*HI) for 24 hours. Pre‐osteoclasts lysed in TRIzol were processed for RNA and subsequent cDNA preparations. QPCR analyses done to assay immune receptor gene expression revealed upregulation of TLR1, TLR2, FcγRIIB, and FcγRIII gene expression levels but not that of TLR4 and FcRγ when compared to *Pg* alone. Bars represent mean ± SE from three independent donors. ^i^ *P* < 0.05 vs *Pg* alone. Note that graphs presented are not on the same scale

Vaccination against *Pg *has been shown to elevate systemic IgG antibody levels and decrease PD, but little is known how the antibodies actually abrogate this disease process.^19^, ^27^, ^28^ We hypothesized that antibodies to *Pg *interfere with the inflammatory nature of the pathogen and inhibit OC formation. Toward this end, pooled human sera with specific antibody titer to *Pg * obtained previously^29^, ^30^, ^31^ were used to assay the effects on OC differentiation and function. PBMC‐derived monocytes were primed with M‐CSF and RANKL prior to introduction of *Pg *alone, or opsonized *Pg *with antibodies (Low or High specific *Pg* ab titer/immune complexes) for 10 days. Quantification of multinucleated OC revealed a significant decrease in osteoclast differentiation in cultures treated with ICs when compared to *Pg* alone (Figure 1B). Furthermore, analyzing resorption pits confirmed the decrease in osteoclast activity following opsonized bacteria/immune complex treatment (Figure 1C). In addition, interaction of *Pg*‐specific antibody with commensals (*Sg*) within the multi‐bacterial PD biofilms had no effect (Figure 1E), which helps to establish the specificity of the antibody response on modulating osteoclast differentiation. We have also assessed mRNA levels in OC following 24 hours treatment with *Pg* or opsonized *Pg*, for classic OC markers ACP5, RANK, CATK, and NFAT. Consistent with the phenotype presented above, treatment of the cells with opsonized bacteria downregulated all the above transcripts with an exception of RANK suggesting suppression of OC differentiation (Figure 2A).

### 3.3 Opsonized bacteria abrogated *P gingivalis* stimulatory effects by modulating immune receptor expression

**Figure 3:**
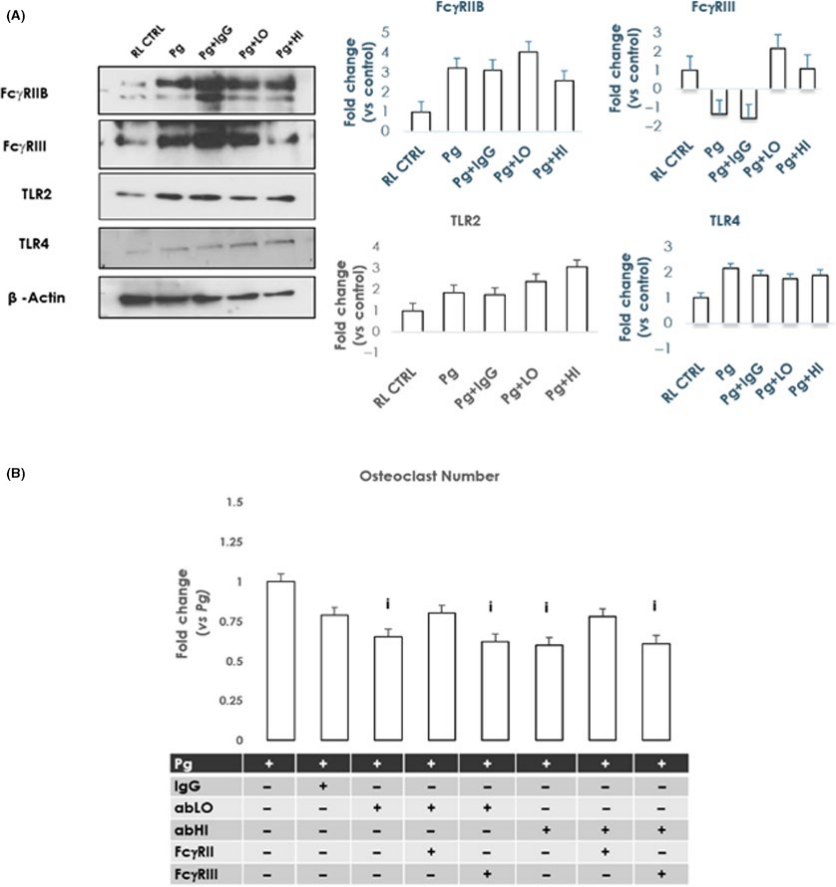
Opsonization of bacteria abrogates *Porphyromonas gingivalis*’ stimulatory effects by modulating immune receptor expression. A, PBMC‐derived monocytes were cultured in the presence of macrophage colony stimulating factor (M‐CSF) and receptor activator of nuclear Factor‐kappa B ligand for 10 d and treated with *Pg* or with opsonized bacteria (*Pg*LO/*Pg*HI) for 48 hours. Osteoclasts lysed in RIPA buffer were processed for total protein. Western blot analyses were done to assay immune receptor expression revealed upregulation of FCγRIIB and FCγRIII, TLR1 and TLR2 expression levels but not that of TLR4 when compared to *Pg* alone. Expression levels were normalized to that of β‐actin. Bars represent mean ± SE from two independent experiments. Note that graphs presented above are not on the same scale. B, Human monocytes were purified from PBMC and were cultured in the presence of M‐CSF and RANKL until binucleation. Pre‐osteoclasts were incubated for 1 h with 2 µg/mL blocking antibodies of FcγRII/FcγRIII/IgG isotype. Subsequently, treated with *P gingivalis* alone (*Pg*) or opsonized bacteria (antibodies to *P gingivalis *complexed with formalin fixed‐*P gingivalis: Pg*LO/*Pg*HI) until multinucleation. Osteoclasts were fixed and stained for TRAcP activity and quantified. Bars represent mean ± SE from two independent cultures. Note reversal of inhibitory effects of opsonized *Pg* following inhibition of FcγRII receptor whereas significant decrease in osteoclast number following application of opsonized bacteria after blocking FcγRIII. ^i^ *P* < 0.05 vs *Pg* alone**. **Bars represent mean ± SE from two independent donors

Members of the TLR family are found on most immune and nonimmune cells, including OC, conceptually to distinguish between host and hostiles.^32^, ^33^, ^34^ Accumulating evidence suggests that activation of the innate immune system may be involved in osteoclast formation^35^ and TLRs are an important family of innate immune receptors.^36^ To address the process of abrogation of osteoclastogenesis by immune complexed *Pg*, we assayed gene expression of known pattern recognition receptors (PRRs) following *Pg* or ICs (*Pg*LO/*Pg*HI) treatment. Gene expression analyses on pre‐osteoclasts using QPCR show upregulation of RANK and TLRs 1, 2, and 4 in the presence of free *Pg*. Messenger RNA levels of TLR2 but not TLR4, FCGRIIB and FCGRIII but not common FCRG were significantly upregulated following treatment with opsonized *Pg* when compared to free *Pg* (Figure 2). Interestingly, the data did not demonstrate a clear dose response with the low vs high‐titer antibody used for opsonizing *Pg* in vitro as might be expected with a simple dilution of an antibody containing sample. Western blot analysis of OC treated with *Pg* either free or opsonized, showed similar TLR2 and TLR4 expression, whereas FcγRIII was significantly upregulated with opsonized *Pg* when compared with *Pg* alone. FcγRIIB protein expression was also upregulated following introduction of *Pg*, but no specific response difference was evident between the treatments (Figure 3A).

### 3.4 **Antibodies to *P gingivalis* inhibit osteoclast differentiation and function induced by the pathogen through Fc**γ**R engagement**

The literature suggests that antibodies against *Pg *can block the interaction of the pathogen with TLR receptors and diminish the activation of the NF‐κB inflammatory pathway, which is highly upregulated by this pathogen in numerous cell types.^37^, ^38^ To determine which FcγR is responsible for the observed osteoclast inhibitory effect of opsonized *Pg*, we introduced neutralizing antibodies against FcγRII and FcγRIII to the pre‐osteoclast cultures. Blocking FcγRII, but not FcγRIII, reversed the effect of opsonized *Pg* suggesting a critical role of this receptor in mediating the inhibitory signaling in osteoclastogenesis (Figure 3B). In contrast, blocking FcγRI had no effect (data not shown). These findings suggested the involvement of immune‐regulatory mechanisms in driving bone loss in chronic periodontal infections.

### 3.5 Antibodies to *P gingivalis* inhibit osteoclast differentiation by inducing a macrophage phenotype

**Figure 4:**
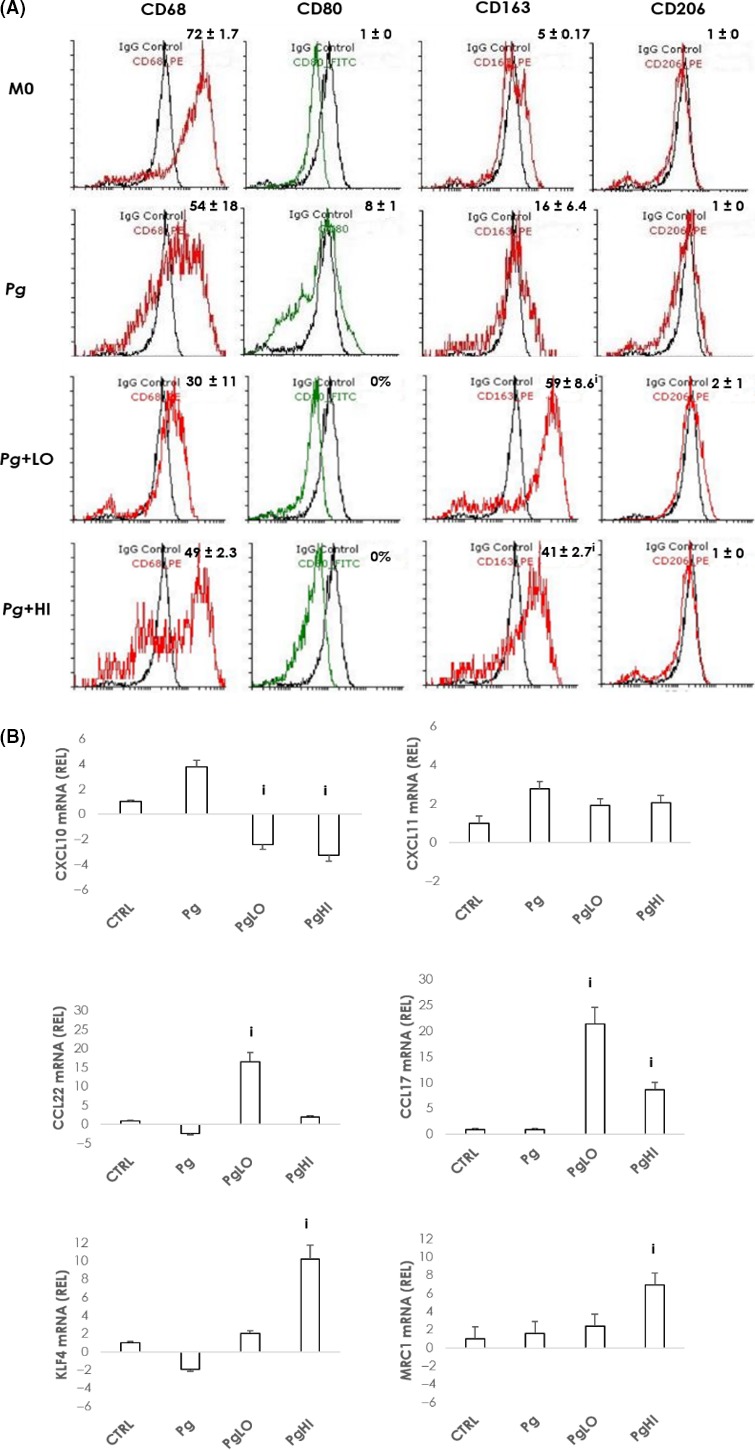
Opsonization of bacteria abrogates *Porphyromonas gingivalis*’ stimulatory effects by driving preosteoclasts to “M2 like” phenotype. A, PBMC‐derived monocytes were cultured in the presence of macrophage colony stimulating factor (M‐CSF) and receptor activator of nuclear Factor‐kappa B ligand for 3 d and treated with *Pg* or with opsonized bacteria (*Pg*LO/*Pg*HI) for 48 h. Cell surface antigens on preosteoclasts were processed for flow cytometry. Briefly, preosteoclasts were rinsed in PBS and detached using PBS‐EDTA. Detached cells were washed in PBS and blocked in PBS‐BSA. Cell surface antibody analysis done to assay macrophage marker expression revealed upregulation of CD68 and CD168 with marked downregulation of CD80 in opsonized *Pg* treated group. Expression levels were normalized to that of IgG Isotype control. Values represent percentage of positive cells (mean ± SE) from two independent donors. ^i^ *P* < 0.05 vs *Pg* alone. The data shown are representative flow cytometry analysis from one of two independent experiments. B, PBMC‐derived monocytes were cultured in the presence of M‐CSF and RANKL for 3 d and treated with *Pg* either free or opsonized (*Pg*LO/*Pg*HI) for 24 h. Pre‐osteoclasts lysed in TRIzol were processed for RNA and subsequent cDNA preparations. QPCR analyses to assay macrophage signature gene expression revealed upregulation of CCL17, CCL22, KLF4, and MRC1 (M2 genes) but not that of CXCL10 and CXCL11 (M1 genes) when compared to *Pg* alone. Bars represent mean ± SE from two independent donors. ^i^ *P* < 0.05 vs *Pg* alone. Note that graphs presented are not on the same scale

Cells of the monocyte/macrophage lineage show considerable plasticity. It is well known that in tissues, mononuclear phagocytes respond to environmental cues and acquire distinct functional phenotypes, such as classical M1‐proinflammatory or alternative M2‐anti‐inflammatory features.^39^, ^40^ Based on these observations, we explored whether opsonized *Pg* can provide sufficient cues for polarization. When pre‐osteoclasts were treated with either free *Pg* or opsonized for 48 hours and phenotypes for macrophage surface markers were assessed, a “M2‐like” phenotype with CD68^high^CD80^low^CD163^high^CD206^low^ signature was evident with the opsonized *Pg* treated cells (Figure 4A). Furthermore, macrophage‐related gene expression was also confirmed by QPCR with marked upregulation of M2 genes (CCL17, KLF4, and MRC1) in the opsonized *Pg* treated cells (Figure 4B). These findings demonstrate a key mechanism of adaptive immune response in PD by providing evidence in vitro and warrants further enquiry to establish the same processes in vivo.

## 4 DISCUSSION

**Figure 5:**
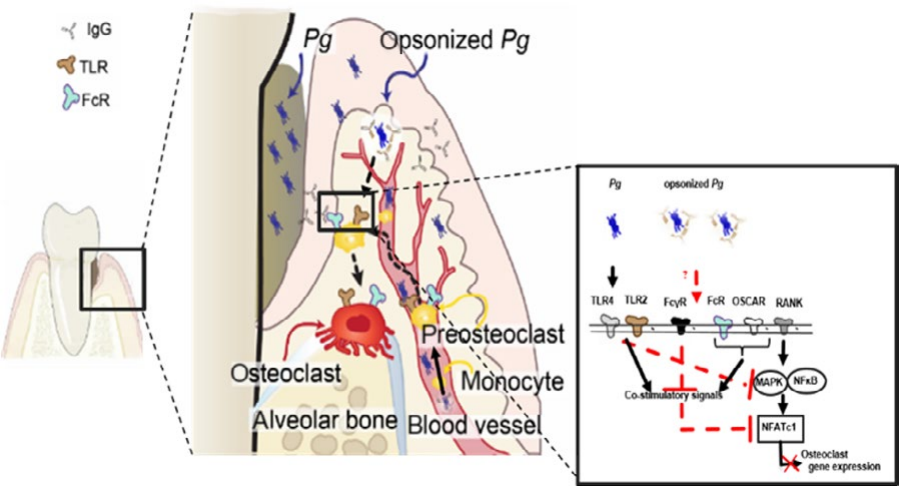
Hypothetical mechanisms of adaptive immune response modulating osteoclastic bone resorption in periodontal diseases. *Porphyromonas gingivalis *both directly, and indirectly through B and T cell stimulation promote osteoclast differentiation and contribute to periodontal bone loss. Interestingly, *Pg *primes antigen specific B cells for antibody production and the IgG antibody response to *Pg *induced by infection/immunization which are delivered to the gingiva by the vasculature, may result in opsonization of *Pg. *Our findings suggest that these opsonized *Pg* block OC differentiation by interacting with TLR and Fc receptors. FcγR being an inhibitory receptor under physiological conditions, is downregulated as osteoclastogenesis proceeds, resulting in an increased cell surface expression of OSCAR due to increased availability of FcRγ. FcRγ, the signaling adaptor molecule for OSCAR, is also shared by IgG‐Fc receptors (FcγRIA and FcγRIII). As influenced by the interaction of the osteoclastic IgG‐Fc receptors with immune complexes (IC), co‐stimulatory signaling (dotted lines) results in inhibition of osteoclastogenesis. Our findings support the idea that opsonized‐*Pg *induce upregulation of inhibitory FcγRs and enhanced TLR signaling, thereby attenuating RANK and co‐stimulatory FcRγ‐induced osteoclastogenesis (dotted lines in red)

Specific adaptive immune responses, with long‐lasting immunity develop following recovery from an infection. It would be expected that adaptive types of responses to the bacterial infection in periodontitis would provide some level of resistance. Thus, the paradigm for this arm of the immune response is that the primary infection triggers these responses leading to immunity and resulting in a state of decreased susceptibility to a subsequent attack by the same organism. The literature has clearly described the characteristics of the humoral adaptive immune response transitioning from gingival health toward various forms of PD with antibodies of all isotypes generally present at low levels in gingival crevicular fluid from healthy sites, with minimal inflammation or tissue disruption.^41^ The literature also supports the existence of local specific antibody production by plasma cells present in inflamed tissues of the periodontal pocket which can be significantly greater than those detected in serum.^42^ Yet, an elevated host response to *Pg* appears to exist with disease and despite high antibody titer the host is apparently unable to clear this chronic infection. Various studies in humans, both cross‐sectional and longitudinal, and in animal models support the idea that antibodies to PD‐associated pathogens can confer protection.^43^, ^44^, ^45^, ^46^ As we have shown previously, nonhuman primates can be vaccinated with *Pg *or selected components resulting in significant increases in local and systemic IgG antibodies leading to decreased clinical disease in a ligature‐induced model.^19^, ^27^, ^28^ As such, numerous studies in rodent, canine, and nonhuman primate models support the finding that active vaccination with *Pg *or its component parts can significantly decrease induced periodontitis.^45^, ^46^, ^47^, ^48^, ^49^ Our findings suggest that the elevated antibodies in humans that occur in response to naturally occurring periodontitis can help to ameliorate this process, potentially through opsonization of oral bacteria, which suppress the osteoclast‐mediated bone loss in periodontitis. This could be explained by the antibody competing with *Pg* as an agonist for PRRs, and selective activation of FcγRs with simultaneous suppression of ITAM receptor FcRγ that regulate bone resorptive processes which are more effective in some patients (Figure 5). Synergistic activation of these TLRs (a type of PRRs) may be responsible for the upregulation of OC differentiation in the presence of *Pg*. Fewer studies have indicated biological activity of ICs. For instance, autoimmune diseases like rheumatoid arthritis show IC deposition in articular joints with soluble antigens and can elicit local inflammatory response leading to pathogenesis.^50^ In addition, ICs also have anti‐inflammatory properties, and this is partly due to their ability to contribute to the generation of regulatory macrophages,^40^, ^51^ which we believe as one of the mechanisms of osteoclast suppression. Recent studies provide evidence that preformed ICs and the intravenous administration of soluble immunoglobulin complexes (IV IgG) can be used to relieve arthritis in experimental models.^52^, ^53^, ^54^ More recently, Staphylococcal Protein A (SPA) administered as a microbial protein‐IgG IC, has been shown to ameliorate antigen‐induced arthritis by modulating pro‐inflammatory cytokines via FcγR.^55^ In a comparable fashion, our data support that IgG antibody responses to periodontal bacteria protect the host from the adverse effects of the infection, potentially through the formation of similar IgG‐bacterial immune complexes (IgG‐ICs) in local tissues and modulate inflammatory bone resorption. But surprisingly, opsonized *Pg* were able to induce an “M2‐like” phenotype in pre‐OC suggesting a key role of adaptive immune response directing the outcome of PD. This study provides data to support the role of humoral immune responses in PDs but the biology of the episodic nature of periodontitis progression and variations in terms of the extent and severity of disease across the population remains unclear.

The literature suggests that *Pg* contributes to the chronic inflammatory lesions of periodontitis, by inducing M1 macrophage population which are usually associated with inflammatory responses to bacterial infections.^56^ While inflammation is required to presage development of adaptive immunity, chronic elevated levels of inflammatory biomolecules in the local tissues are associated with undermining epithelium integrity, connective tissue degradation, and activation of osteoclastogenesis leading to alveolar bone loss.^57^, ^58^ However, a collateral aspect of the induction of M1 cells, particularly through engagement of TLRs, is signaling through the NFκB pathway and production of an array of pro‐inflammatory mediators.^59^ We have previously shown that *Pg *clearly has the ability to trigger this pathway in macrophages and synergize with host factors, that is, IFNγ and extrinsic LPS to induce significant elevations in M1‐produced inflammatory mediators.^60^ Recent findings also suggest that *Pg* selectively tolerate macrophage subsets that could facilitate immunopathology and marginalize immunity.^61^ The importance of macrophages in alveolar bone resorption elicited by *Pg *infection in mice has also emphasized the profile of periodontal infiltrating macrophages to be dominantly an M1 type cell.^62^ Evidence exists to demonstrate that *Pg‐*LPS alone can weakly activate macrophage polarization, while inducing pro‐inflammatory mediators via TLR2 engagement.^63^ These findings are consistent with our observations regarding the capacity of *Pg *to induce a specific response profile in macrophages with some preference for polarizing toward an M1 phenotype.^60^ The finding that was rather unexpected was the apparent capacity of opsonized *Pg *to downregulate/block activation of OC markers and transform them to an M2‐like phenotype as evidenced by surface markers (CCL17 and MRC1) that would help engage immunoregulatory cells and adaptive immunity.

We choose to utilize pooled human sera derived from subjects identified as periodontally healthy or those with a range of extent/severity of periodontitis thereby allowing us to construct low and high experimental antibody preparations.^29^, ^31^ This strategy allowed us to focus on the relationship of antibody level acquired during colonization/infection with *Pg *in humans. However, we recognize that these antibody populations derived from different individuals also likely differ in subclass distribution, avidity, and antigen specificity that could impact the experimental outcomes, although there is data identifying these types of antibody variations driving differences in function. Further studies are warranted to assess if these findings are specific characteristics of opsonized *Pg* alone or a more general pattern of humoral immune response to oral pathogenic bacteria.

In conclusion, we provide evidence to support a bone protective role of antibodies in opsonizing *Pg* to produce ICs that modify osteoclast differentiation and activity. Our findings suggest a potential competition among *Pg* and opsonized complexes as agonists to PRRs, and through selective activation of FcγRs and simultaneous suppression of ITAM receptor FcRγ to regulate bone resorption. Our findings also extend the concept that this type of variation in antibody function within the population may help to explain disease resistance or extent/severity and that *Pg *immunization‐induced antibodies could effectively decrease OC activities. Further investigations will be necessary to compare the effectiveness of naturally occurring antibody generated through infection/disease, and actively induced immune antibodies to establish the characteristics of the adaptive immune response in regulating OC functions in periodontitis.

## CONFLICT OF INTEREST

Authors report no conflicts of interest related to this study.

## AUTHOR CONTRIBUTIONS

SP contributed to the experimental design and study activities, interpretation of data, and prepared the manuscript. JE contributed to the experimental design, oversight of the conduct of the study, and contributed to content of the manuscript. SH contributed to the experimental design, study activities, and spearheaded the preparation of the manuscript.
